# New Insights on Vitamin K Metabolism in Senegalese sole (*Solea senegalensis*) Based on Ontogenetic and Tissue-Specific Vitamin K Epoxide Reductase Molecular Data

**DOI:** 10.3390/ijms21103489

**Published:** 2020-05-15

**Authors:** Silvia Beato, Carlos Marques, Vincent Laizé, Paulo J. Gavaia, Ignacio Fernández

**Affiliations:** 1 Campus de Vegazana, Universidad de León (ULE), s/n, 24071 León, Spain; silviabeato@hotmail.it; 2 Centro de Ciências do Mar (CCMAR), Universidade do Algarve, Campus de Gambelas, 8005-139 Faro, Portugal; cd.marques@outlook.pt (C.M.); vlaize@ualg.pt (V.L.); pgavaia@ualg.pt (P.J.G.); 3 Departamento de Ciências Biomédicas e Medicina (DCBM), Universidade do Algarve, Campus de Gambelas, 8005-139 Faro, Portugal; 4 Center for Aquaculture Research, Agrarian Technological Institute of Castile and Leon, Ctra. Arévalo, S/n. Zamarramala, 40196 Segovia, Spain

**Keywords:** Vitamin K, gene expression, evolution, flatfish, nutrition, pollution

## Abstract

Vitamin K (VK) is a key nutrient for several biological processes (e.g., blood clotting and bone metabolism). To fulfill VK nutritional requirements, VK action as an activator of pregnane X receptor (Pxr) signaling pathway, and as a co-factor of γ-glutamyl carboxylase enzyme, should be considered. In this regard, VK recycling through vitamin K epoxide reductases (Vkors) is essential and should be better understood. Here, the expression patterns of *vitamin K epoxide reductase complex subunit 1* (*vkorc1*) and *vkorc1 like 1* (*vkorc1l1*) were determined during the larval ontogeny of Senegalese sole (*Solea senegalensis*), and in early juveniles cultured under different physiological conditions. Full-length transcripts for *ssvkorc1* and *ssvkorc1l1* were determined and peptide sequences were found to be evolutionarily conserved. During larval development, expression of *ssvkorc1* showed a slight increase during absence or low feed intake. Expression of *ssvkorc1l1* continuously decreased until 24 h post-fertilization, and remained constant afterwards. Both *ssvkors* were ubiquitously expressed in adult tissues, and highest expression was found in liver for *ssvkorc1*, and ovary and brain for *ssvkorc1l1*. Expression of *ssvkorc1* and *ssvkorc1l1* was differentially regulated under physiological conditions related to fasting and re-feeding, but also under VK dietary supplementation and induced deficiency. The present work provides new and basic molecular clues evidencing how VK metabolism in marine fish is sensitive to nutritional and environmental conditions.

## 1. Introduction

Increasing a sustainable animal production is essential to warrant world food security and nutrition in the nearest future [1,2]. Animal protein sources of aquatic origin are generally healthier (richer in n-3 and n-6 polyunsaturated fatty acids [3]), cheaper to produce and more sustainable (with better feed conversion rates) than those from the terrestrial livestock [4]. Several factors determine the world production of animal protein of aquatic origin. On the one hand, the loss of natural habitats, its degradation and pollution, and the reduction of biodiversity, reduce the productivity and profitability of ecosystem services and affect the well-being of human population [5,6]. On the other hand, the aquaculture sector, representing more than 50% of the total fish production, largely depends on genetic selection, husbandry practices and nutrition [1]. Regarding nutrition, fulfilling nutritional requirements is a cornerstone for aquaculture development [7,8], being critical for fish survival, growth potential, feed conversion efficiency and product quality, mainly during early development (i.e., embryonic, larval and early juvenile stages).

Nutritional requirements for micronutrients (minerals and vitamins) have still to be fine-tuned for marine fish larvae [9]. Their amount and chemical form depend on factors such as fish species, developmental phase, physiological stage, environmental conditions and nutritional approach used, among others (reviewed in [10]). Regarding vitamin K (VK), our understanding of VK metabolism and VK-dependent molecular pathways has increased during the last decade (reviewed in [10,11,12,13]). Three chemical VK forms exist: phylloquinone (VK1, from vegetable origin), menaquinones (VK2 or MK, from bacterial or animal origin) and menadione (VK3, synthetic). VK requirements are strongly dependent on the chemical form and fish species considered, ranging from 0.1 mg to 20 mg kg^−1^. In this sense, although VK3 has a limited bioavailability, it is the most common source of VK in aquafeeds (reviewed in [12]). In general, VK nutritional requirements were considered to be low, as VK was thought to be mainly involved in blood coagulation, and no major signs of bleeding disorders were observed in fish with low VK dietary levels [12]. In fish species, the key role of VK in biological processes, such as sex hormone synthesis/release and reproductive performance [14], skeletal development and maintenance [15,16,17,18], neural development and cognitive capacities [19], and redox system homeostasis, vasculogenesis and visual phototransduction [18], has been evidenced, and higher VK nutritional requirements during early developmental stages than previously thought were suggested [15,16,18].

For a proper estimation of VK nutritional requirements throughout larval development, a detailed molecular characterization of the main VK gene networks and pathways, and how they respond under changing physiological conditions, is fundamental. VK exerts its biological functions through two main pathways: as an agonist of the pregnane X receptor (Pxr), which has transcriptional activity [20,21], and as a co-factor of the γ-glutamyl carboxylase (Ggcx) enzyme, which promotes the conversion of glutamate (Glu) into γ-carboxyglutamate (Gla) residues in VK-dependent proteins (VKDPs; reviewed by Oldenburg et al. [22]). Carboxylation requires the abstraction of a proton from the 4-carbon of the glutamate residue by reduced VK and results in the conversion of VK into VK epoxide. The VK epoxide must be recycled into VK before it can be reused, a reaction that is catalyzed by two VK 2,3-epoxide reductases (Vkors), the Vkor complex subunit 1 (Vkorc1) and Vkorc1-like 1 (Vkorc1l1), which probably arose from the duplication of a Vkor ancestor gene [23,24,25,26,27,28].

Senegalese sole (*Solea senegalensis*) is a marine flatfish used to understand the molecular mechanisms of asymmetrical morphology [29,30], to conduct biomonitoring programs of coastal ecosystems [31,32], and is an important fish species for European aquaculture diversification [33]. As a result of the interest of both the industry and academia, the knowledge on optimal husbandry practices and environmental conditions, genetic background, and/or nutrition, has recently been improved (reviewed in Muñoz-Cueto et al. [34]). Nevertheless, its production has been hampered by problems in the reproduction of F1 broodstocks [14,35,36,37,38] and the high incidence of skeletal deformities [39,40,41,42,43,44,45], among other factors.

Early during their development, Senegalese sole larvae undergo metamorphosis, a highly complex transition process that involves deep morphological, biochemical and physiological transformations that allow pelagic larvae to become juveniles with a benthic behavior [46]; a phase where the onset of many skeletal deformities might occur [45]. Indeed, Senegalese sole larval performance and skeletal quality improvement through dietary VK1 supplementation was previously suggested [15]. We also observed Senegalese sole *pxr* gene expression dependent on the developmental-stage and tissue considered, suggesting a variable VK requirement for its ligand transactivation [19]. Here we hypothesized that *vkorc1* and *vkorc1l1* gene expression might be altered through larval development and will be tissue-specific, reflecting that dietary VK requirement might depend on fish development and biological process considered. The present work aims at getting full-length transcript sequences of Senegalese sole *vkors*, at studying their evolutionary conservation, and at determining their expression patterns during larval development and adult tissues, but also under relevant physiological conditions, in order to understand how VK recycling is vital to promoting good growth and normal development in fish.

## 2. Results

### 2.1. Senegalese Sole vkorc1 and vkorc1l1 Full-Length cDNA Sequences and Common Protein Sequence Features with Vertebrate Vkors

**Figure 1:**
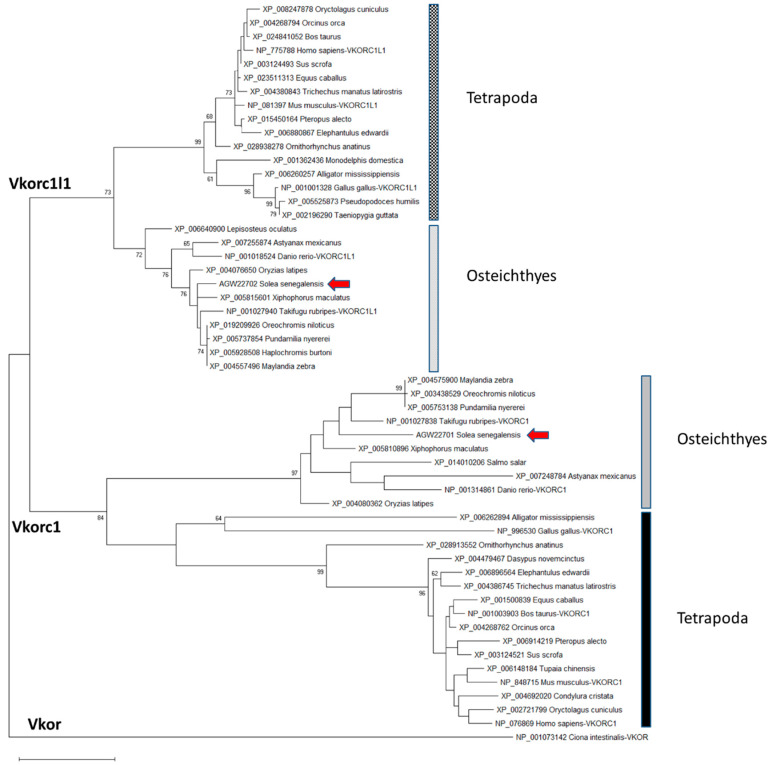
Maximum likelihood phylogenetic tree of vitamin K epoxide reductases (Vkors). The evolutionary history was inferred by using the Maximum Likelihood method and Le and Gascuel model [47]. The percentage of trees in which the associated taxa clustered together is shown next to the branches. Initial tree(s) for the heuristic search were obtained by applying the Neighbor Joining method to a matrix of pairwise distances estimated using a Jones-Taylor-Thornton (JTT) model. A discrete Gamma distribution was used to model evolutionary rate differences among sites (5 categories (+G, parameter = 0.7756)). The tree is drawn to scale, with branch lengths measured in the number of substitutions per site. This analysis involved 54 amino acid sequences. All positions with less than 95% site coverage were eliminated, i.e., fewer than 5% alignment gaps, missing data, and ambiguous bases were allowed at any position (partial deletion option). There was a total of 150 positions in the final dataset. Evolutionary analyses were conducted in MEGA X [48]. *Ciona intestinalis* Vkor sequence was used as outgroup to root the tree. Scale bar represents nº of substitutions per site.

Partial nucleotide sequences for Senegalese sole *vkors* published by Richard et al. [15]—a 489 bp fragment for *vkorc1l1* and a 201 bp fragment for *vkor-1—*were used to get 5’ and 3’ transcript ends by Rapid Amplification of Complementary deoxyribonucleic acid (cDNA) Ends (RACE) polymerase chain reaction (PCR). Using available cDNA fragments, the full-length transcripts for *ssvkorc1l1* (1168 bp; GenBank accession no. KC108910) and *ssvkor-1* (748 bp; GenBank accession no. KC108911) were reconstructed and later confirmed by PCR. Deduced peptides ssVkorc1l1 (GenBank accession no. AGW22701) and ssVkorc1 (GenBank accession no. AGW22702) were 175 amino acids (aa) and 161 aa long, respectively. The phylogenetic analysis of 54 vertebrate Vkor protein sequences (see species and GenBank accession no. in Table S1) revealed that AGW22701 and AGW22702 clustered with annotated sequences of Vkorc1 and Vkorc1l1, respectively, and had highest homology with teleost sequences, confirming their identity as Senegalese sole orthologues ssVkorc1 and ssVkorc1l1, respectively (Figure 1).

**Figure 2:**
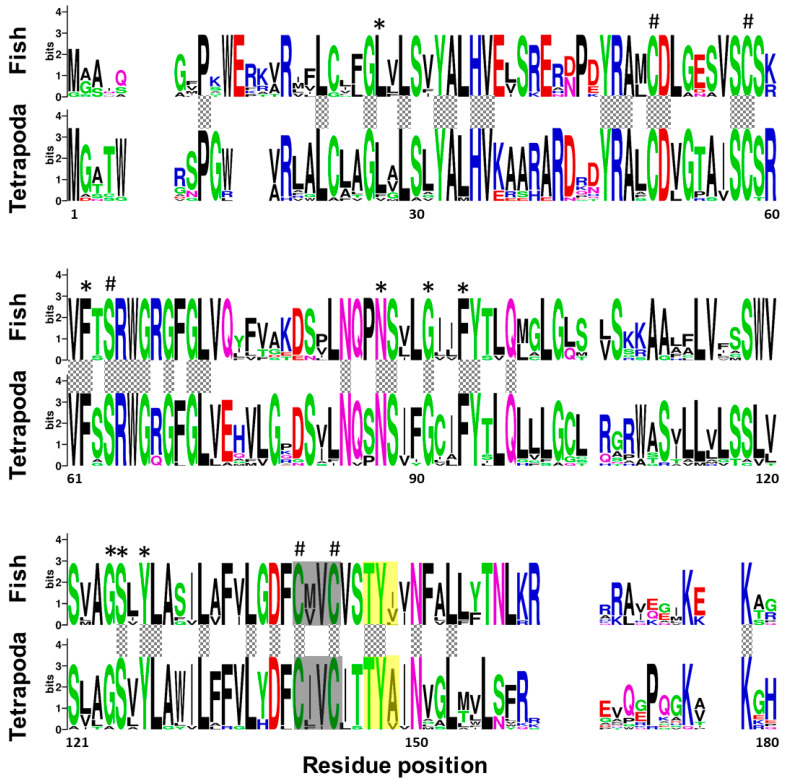
Conservation of amino acid residues in vitamin K epoxide reductase complex subunit 1 (Vkorc1) between fish and tetrapods. Logos were constructed from isoform-specific alignments of mature peptides. The height of the letters is directly proportional to their frequency. Asterisks indicate amino acids fully conserved within fish species; hashes indicate Vkor active sites (four cysteines and one serine); yellow box, warfarin binding motif (TYX); grey box, hydrophobic environment of the thiol redox site of the enzyme; grey squared boxes between alignments, amino acids with full conservation between both taxa: fish and tetrapoda.

A multi-sequence alignment, using T-coffee platform, of 7 protein sequences corresponding to Vkor sequence from sea squirt (*Ciona intestinalis*), and Vkorc1 and Vkorc1l1 sequences from Senegalese sole, human (*Homo sapiens*) and mouse (*Mus musculus*), showed a high degree of conservation of the characteristic four cysteines and the serine residues, the four transmembrane domains, the thiol redox (CXXC) site, and the warfarin binding motif (TYX; Figure S1). A weblogo comparative analysis of fish and tetrapod Vkorc1 and Vkorc1l1 peptides also revealed the conservation of important amino acids related to Vkors protein function in a broader context. Fish and tetrapod Vkorc1 protein sequences (Figure 2) have 44 amino acid residues fully conserved, including the previously reported and characteristic four cysteines and the serine, as well as the CXXC site of known hydrophobic environment. Fish Vkorc1 protein sequences showed a higher conservation degree than tetrapods, with 99 amino acid residues fully conserved. Furthermore, in both taxonomic groups the warfarin binding motif was identified, although not fully conserved, with a lower degree of conservation on the third amino acid residue in fish species. 

**Figure 3:**
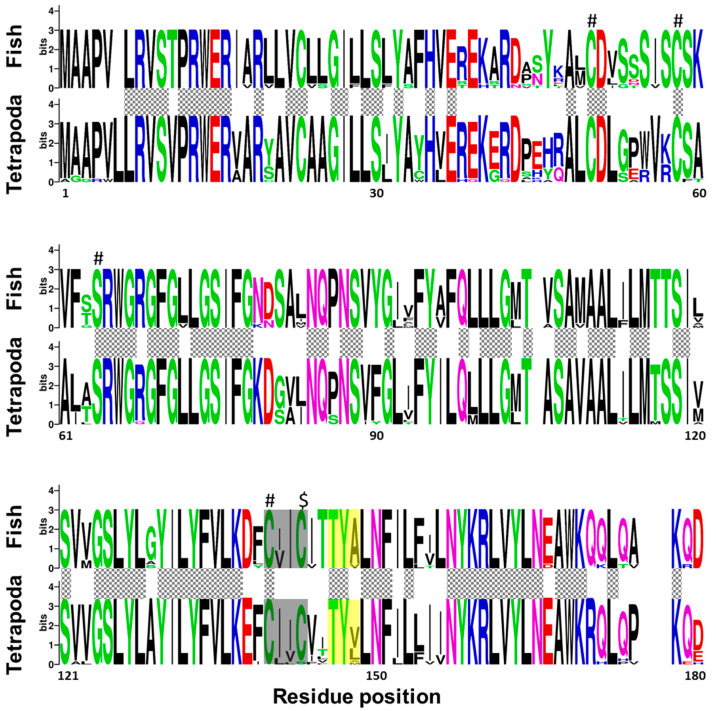
Conservation of amino acid residues in vitamin K epoxide reductase complex subunit 1 like 1 (Vkorc1l1) between fish and tetrapods. Logos were constructed from isoform-specific alignments of mature peptides. The height of the letters is directly proportional to their frequency. Please, note that: Hashes indicate Vkor active sites (three cysteines and serine); dollar symbol, indicates the non-fully conserved Vkor active site (a cysteine); yellow box, warfarin binding motif (TYX); grey box, the known hydrophobic environment of the thiol redox site of the enzyme; grey squared boxes between alignments, amino acids with full conservation between both taxa: fish and tetrapoda.

Regarding fish and tetrapod, Vkorc1l1 peptides revealed that 92 amino acid residues were fully conserved between the two taxonomic groups, and 130 among fish species (Figure 3). In contrast to Vkorc1, and although the reported characteristic serine was fully conserved, only three of the four cysteines were fully conserved, being not fully conserved in the last one of the thiol redox (CXXC) site. As for Vkorc1, warfarin binding motif (TYX) was not fully conserved in Vkorc1l1. In contrast to Vkorc1, the third residue of the TYX motive was less variable in fish species. 

### 2.2. Expression of ssvkorc1 and ssvkorc1l1 During Development and in Adult Tissues

**Figure 4:**
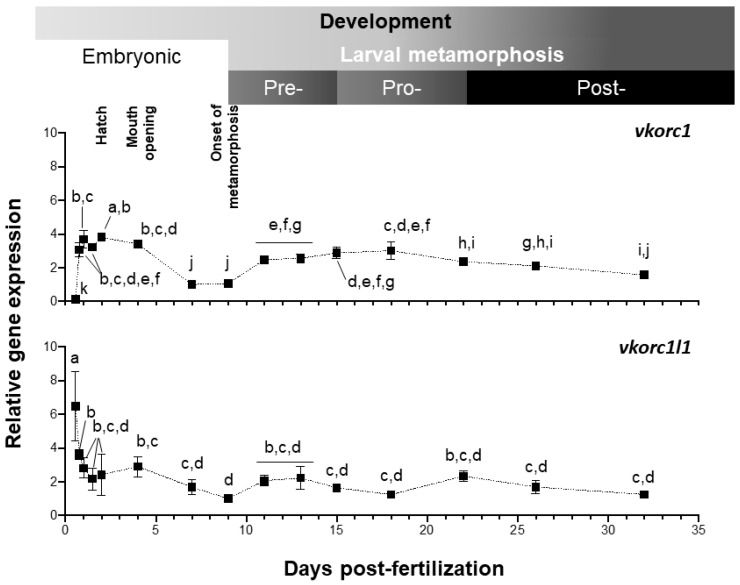
Relative gene expression of Senegalese sole *vitamin K epoxide reductase complex subunit 1* (*ssvkorc1*) and *vkorc1 like 1* (*ssvkorc1l1*) throughout larval development. Transcript levels of *ssvkorc1* (upper image) and *ssvkorc1l1* (bottom image) were determined by qPCR and normalized using *ubiquitin* (*ubq*) gene expression. Levels at 7 and 9 hours post-fertilization (dpf) were used as reference and set to 1 for *ssvkorc1* and *ssvkorc1l1*, respectively. Different letters over gene expression values denote significant differences among developmental stages (n = 3; one-way analysis of variance (ANOVA), Tukey multiple-comparison test; *P* < 0.05).

Expression of Senegalese sole *vkor* genes was evaluated by quantitative PCR (qPCR), from 13.5 h post-fertilization (hpf) embryonic stage, to 32 days post-fertilization (dpf) post-metamorphic juvenile stage (Figure 4). Both genes showed a clearly distinct pattern of expression along larval development. Level of *ssvkorc1* expression was the highest (up to 3.8 folds) after hatching (48 hpf), diminished at 7–9 dpf (1.0–1.1 folds), increased through pre- and pro-metamorphosis (up to 3.0 folds) and remained stable during post-metamorphosis (ranging from 1.5 to 2.3 folds). *ssvkorc1l1* expression pattern during larval development and metamorphosis showed an initial sharp decrease, from the maximal gene expression at 13.5 hpf (6.5 folds) to a 50% decreased level at 18.5 hpf (3.6 folds). Afterwards, *ssvkorc1l1* expression remained constant (between 2.2 and 2.8 folds), decreased to its minimal expression at 9 dpf (1.0 folds), and slightly increased to remain constant during pre-, pro- and post-metamorphosis (around 1.2 to 2.3 folds).

**Figure 5:**
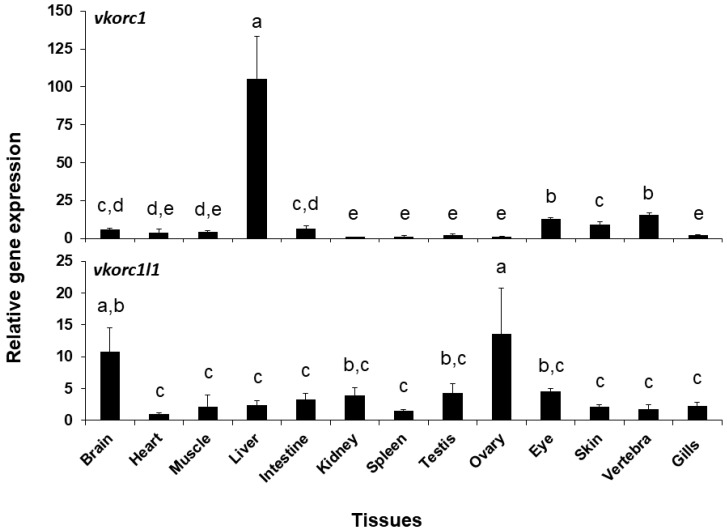
Relative gene expression of Senegalese sole *vitamin K epoxide reductase complex subunit 1* (*ssvkorc1*) and *vkorc1 like 1* (*ssvkorc1l1*) in adult tissues. Transcript levels of *ssvkorc1* (upper image) and *ssvkorc1l1* (bottom image) were determined by qPCR and normalized using *ubiquitin* (*ubq*) gene expression. Levels in kidney and heart were used as reference and set to 1 for *ssvkorc1* and *ssvkorc1l1*, respectively. Different letters on the top of each bar denote significant differences among tissues (n = 3; one-way ANOVA, Tukey multiple-comparison test; *P* < 0.05).

Expression of *ssvkorc1* and *ssvkorc1l1* was also different depending on the tissue considered (Figure 5). A clearly high expression of *ssvkorc1* was found in the liver (105.4 ± 28.1 folds), while it was low in the other tissues (from 1.0 to 13.2 folds). A more homogeneous gene expression pattern was observed for *ssvkorc1l1*. In this case, highest expression levels were found in brain and ovary tissues (10.7 and 13.5 folds, respectively), while intermediate gene expression values were found in kidney, testis and eye tissues (from 3.8 to 4.6 folds) and lowest in the other explored tissues (from 1.0 to 3.2 folds). A direct comparison of the contribution of both genes (comparing cycle threshold (Ct) values of both genes for each tissue; Table S2) shows that *ssvkorc1l1* gene expression levels (Ct values ranging from 33 to 37) are lower than those of *ssvkorc1* (with Ct values ranging from 26 to 31) in all tissues unless in the ovary, where both genes might almost equally contribute (Ct values ranging 29–31 and 28–29, respectively).

### 2.3. Expression of ssvkorc1 and sskorc1l1 under Different Physiological Conditions

**Figure 6:**
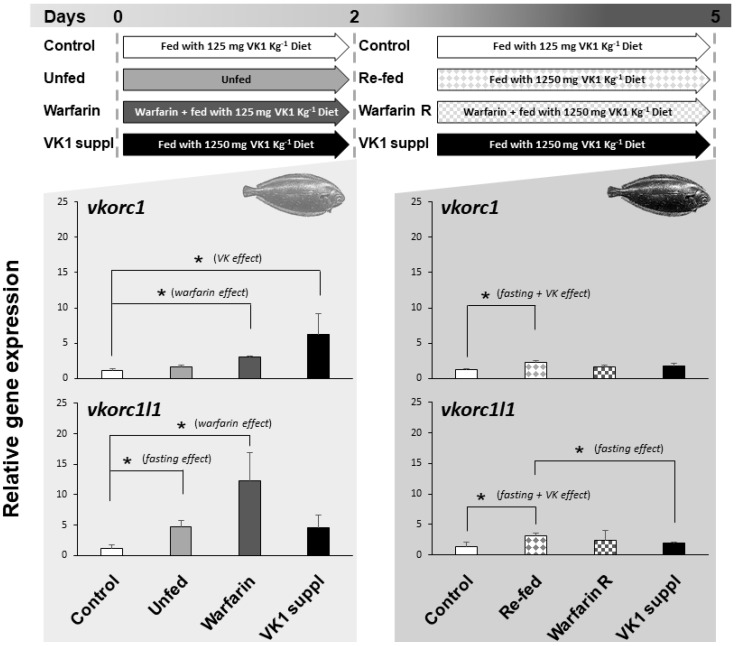
Experimental design and relative gene expression of Senegalese sole *vitamin K epoxide reductase complex subunit 1* (*ssvkorc1*) and *vkorc1 like 1* (*ssvkorc1l1*) in juveniles cultured under different physiological conditions. Top image: experimental design indicating the physiological conditions to which Senegalese sole juveniles were subjected, from zero to two days and from two to five days. Grey shadow graphs: mean and standard deviations values of *ssvkorc1* (upper image) and *ssvkorc1l1* (bottom image) transcript levels at two and five sampling days. Transcript levels were determined by qPCR and normalized using *ubiquitin* (*ubq*) gene expression levels. Levels in Control group were used as reference and set to 1. Left side of the graphs: juveniles fed with 125VK1 diet (Control), kept unfed (Unfed), exposed to 25 mg L^−1^ of warfarin while fed with 125VK1 diet (Warfarin), or fed with 1250VK1 diet (VK1 suppl) for two days. Right side of the graphs: juveniles fed with 125VK1 diet for five days (Control), fed with 1250VK1 diet for three days after being kept unfed for two days (Re-fed), fed with 1250VK1 diet for three days while being exposed to 25 mg L^−1^ of warfarin (Warfarin R), or fed with 1250VK1 diet (VK1 suppl) for five days of experiment. At two days all Experimental groups were compared to Control group, while at five days all experimental groups were compared to Control and VK1 suppl group. Asterisks on the top denote significant differences between the two experimental groups compared (n = 3; Student’s t-test; *P* < 0.05). Text in brackets indicates the effect studied within each comparison.

The expression of *ssvkorc1* and *ssvkorc1l1* in whole Senegalese sole early juveniles, reared under relevant physiological conditions, and found in the natural environment and/or specific to fish farming environment (fasting, re-feeding, presence of emerging contaminants or supplementation of VK), was evaluated (Figure 6). Expression of *ssvkorc1* in early juveniles unfed for two days did not show significant differences with control fish (fish fed with 125 mg kg^−1^ dry weight (DW) of phylloquinone (VK1); 125VK1 diet), ranging 1.2 ± 0.2 to 1.6 ± 0.3 folds. Nevertheless, juveniles fasted for two days and then re-fed for three days with a feed supplemented with VK1 (1250 mg kg^−1^ DW of VK1; 1250VK1 diet)—compiling fasting and VK effects—showed an increased *ssvkorc1* expression (2.2 ± 0.3 folds) compared to control fish (fed with 125VK1 diet). In contrast, *ssvkorc1l1* expression was up-regulated (4.8 ± 0.9 folds) in unfed juveniles during two days compared to the control group. Furthermore, when unfed fish were re-fed for three days with 1250VK1 diet, *ssvkorc1l1* expression was significantly higher (3.2 ± 0.4 folds) than that of fish fed 125VK1 diet (Control) and 1250VK1 diet (VK1 suppl) over five days.

When juveniles fed 125VK1 diet were exposed for two days to 25 mg L^−1^ of warfarin (Warfarin group), a rodenticide commonly used worldwide that induces VK deficiency, gene expression of *ssvkorc1* (3.0 ± 0.1 folds) and *ssvkorc1l1* (12.2 ± 4.6 folds) was significantly increased. After these two days, continued warfarin exposure but feeding with 1250VK1 diet led expression of *ssvkorc1* and *ssvkorc1l1* to reach levels not significantly different from either Control or VK1 supplied groups at five days.

Finally, expression of *ssvkorc1* and *ssvkorc1l1* in juveniles fed with 1250VK1 diet (10 times more VK1 than the Control diet) showed a different regulation throughout time. While *ssvkorc1* expression was up-regulated (6.2 folds) after two days, it returned to basal levels after five days (1.8 ± 0.3 *versus* 1.2 ± 0.2 folds in Control group). The tendency for *ssvkorc1l1* expression was similar after two days (an increase of 4.5 ± 2.1 folds *versus* 1.2 ± 0.5 folds in the Control group), although differences were not significantly different. After five days of feeding with 1250VK1 diet, expression was also unaltered (1.9 ± 0.2 *versus* 1.4 ± 0.7 folds in Control group).

## 3. Discussion

Vitamin K (VK) is an essential micronutrient that vertebrates do not synthesize, thus they depend on dietary sources to obtain the required daily amounts. Nowadays, VK is known to be required for blood coagulation [49,50], skeletal tissue [21] and redox [23,51] homeostasis, sphingolipid [52] and glucose metabolism [53], neural development and cognitive capacities [52,54,55,56], pathological calcification and inflammation [57,58,59], angiogenesis [60], and reproduction [61,62,63]. Studies in fish also suggest that VK is required for blood coagulation [16,64], skeletal development and skeletal tissues homeostasis [15,17,18], redox homeostasis [18], sphingolipid metabolism [14], brain development and cognitive capacities [19], pathological calcification and inflammation [16], and reproduction [14,16,65], reinforcing the idea of a well conserved function of VK throughout vertebrate evolution [26,66]. Nevertheless, the identification and characterization of the different molecular pathways where VK acts in vertebrates development and physiology, particularly how they respond to relevant physiological conditions, still needs to be determined in order to identify potential suitable biomarkers of VK nutritional status [67,68,69]. 

### 3.1. ssvkorc1 and ssvkorc1l1 are Orthologous to Vertebrate Vkors

Up to date, Vkor orthologues have been detected in different taxa of eukaryotic organisms, being Vkorc1 and Vkorc1l1 paralogues that probably originated through a duplication event occurring at the base of the vertebrate split from other metazoans [66]. While Goodstadt and Ponting [70] were the first to identify the four conserved cysteines and a conserved serine/threonine as residues likely required for the enzymatic activity of Vkorc1, by comparing Vkorc1 sequences from 37 species of archaea, eubacteria, plants, invertebrates and vertebrates, we and others further confirmed the conservation of these five residues in other species [65,66]. Here, we have cloned two *vkor*-related transcripts in Senegalese sole (*ssvkorc1* and *ssvkorc1l1*, respectively), and confirmed that related peptides are orthologous to vertebrate Vkorc1 and Vkorc1l1 through phylogenetic analysis. Senegalese sole Vkorc1 and Vkorc1l1 showed the characteristic cysteine and serine conserved residues, the four transmembrane domains, the thiol redox site and the warfarin binding motif characteristic of Vkors. In addition, we have identified the presence of highly conserved residues not only among fish species, but also when comparing sequences from fish and tetrapod taxa, and evidenced a higher amino acid residue conservation in Vkorc1l1 than in Vkorc1. Conserved residues have been associated with the VK 2,3-epoxide to VK quinone, and VK quinone to VK quinol reduction (the above-mentioned four cysteines and the serine), the transmembrane helices, and the putative functional residues essential for quinone substrate reduction or substrate binding and specificity [66,71]. In this sense, some of the residues reported to be important in K vitamers binding with human Vkorc1 [71] were fully conserved in fish species. For instance, residues F55, N80 and F87 (but not F83 and L112), involved in VK1 MK-4 and MK-7 binding, and residues A115 and G116 for MK-7 binding, were conserved. We also confirmed the higher conservation of the warfarin binding motif (TYX) in fish Vkorc1l1 proteins, with most species exhibiting the fully conserved form (TYA motif) in human and mouse Vkorc1, as previously reported in zebrafish (*Danio rerio*) Vkors [65]. Research studies on protein sequence analysis and functional characterization of Vkors in fish species are scarce compared to the extensive effort made in mammals, [72,73,74,75] and more recently in birds [76]. Taking into account the relevant roles of Vkorc1 and Vkorc1l1 in recycling VK, and thus on vertebrate development and physiology, it may be highly relevant for the aquaculture industry to identify single nucleotide polymorphisms (SNPs) in *vkors* from important species (including Senegalese sole), toward the selection of breeders with lower VK nutritional requirements and/or more efficient VK recycling. The present research work providing the cDNA sequences might also be used for the recombinant production of ssVkorc1 and ssVkorc1l1 in in vitro systems, to assess their function and characterize their ability to bind and recycle different K vitamers. This would certainly help to identify the most suitable source of VK (or the best combination) to be included in the diets for each particular fish species.

### 3.2. ssvkorc1 and ssvkorc1l1 Distinct Gene Expression Patterns May Reflect Their Functional Specialization

The exploration of the gene expression values of Senegalese sole *vkors*, by means of qPCR, might be a suitable approach to get insights into the nutritional requirements of VK along its ontogenetic development, and in different tissues/organs. The potential effects of organ size being variable along the larval development (and thus, the portion of its RNA with respect to the total RNA) on the interpretations performed using a whole-body RNA extraction protocol might not be neglected. Nevertheless, the higher expression of *ssvkorc1* and *ssvkorc1l1* during embryonic development (from 18.5 hpf to 2 dpf and from 13.5 to 24 hpf, respectively) is consistent with the expression profile of both genes in zebrafish [65], suggesting that VK is actively recycled during fish endotrophic development, due to the lack of new dietary incomes of VK. These high values are also consistent with a higher developmental and physiological impact of VK deficiency, induced by warfarin exposure during zebrafish embryogenesis [18], than during larval development and/or adult stages [16,17]. These results are clearly in line with the most known consequences of VK deficiency: the preterm death by excessive bleeding and abnormal skeletal development in newborns [13,77,78,79]. Furthermore, increased gene expression of *ssvkorc1* from post-fertilization to mouth opening, in parallel with a decreased expression of *ssvkorc1l1*, is in agreement with the restricted capacity of Vkorc1l1 in supporting VKDP carboxylation in liver and bone only during the mammalian pre- and perinatal periods in the absence of Vkorc1 [27]. A second peak of expression was observed for *ssvkorc1* during pre- and pro-metamorphosis. When Senegalese sole larvae initiate hormonal and morphological transformations in order to adapt their body to a benthonic way of life, a stressful condition that correlates with reduced feed intake [45,80], an increased VK recycling through Vkorc1 might help to sustain VKDPs hepatic γ-carboxylation.

Vertebrate genomes contain two Vkors, Vkorc1 and its paralog Vkorc1l1 [26]. Upon gene duplication, one of the paralogs can evolve a new function, a process known as neofunctionalization. In this regard, patterns of *ssvkorc1* and *ssvkorc1l1* expression were also different in the adult tissues selected in this work. Highest and almost exclusive *ssvkorc1* expression in liver is consistent with expression data reported for *vkorc1* in mouse liver [24], and functional data showing that Vkorc1 is the main isoform responsible for recycling VK epoxide originated from hepatic γ-carboxylation of hemostasis-related VKDPs [25]. Vkorc1l1 has been recently shown to support in vivo VKDP carboxylation in liver and bone during the pre- and perinatal periods in the absence of Vkorc1 [27,28], suggesting a partial redundancy between both Vkors. In line with this, *ssvkorc1* and *ssvkorc1l1* were here found to be expressed in both tissues (liver and vertebra). High expression of *ssvkorc1l1* in brain is in agreement with previous reports, showing high expression in fish and rodent brains [24,65,81], and the proposed role of VK in brain development and homeostasis [19,52]. High expression of *ssvkorc1l1* observed in ovary further supports a central role of VK in reproduction [14,82], and is in agreement with the VK nutritional requirement proposed for offspring development [83]. The tissue gene expression patterns here reported suggest an urgent need for determining the VK nutritional requirements for the normal development and functioning of neural and reproductive systems, most probably with K2 vitamers.

### 3.3. Expression of ssvkorc1 and ssvkorc1l1 is Differentially Regulated under Relevant Physiological Conditions

In general, and in contrast to the other fat-soluble vitamins, vertebrates seemed to have a low VK nutritional requirement [10]. Recommended dietary levels are in the order of 20,000 to 50,000 international units (IU) kg^−1^ for vitamin A, 240 to 1,300,000 IU VD_3_ kg^−1^ for vitamin D, and 25 to 3000 mg α-tocopherol kg^−1^ for vitamin E, depending on the fish species considered [10], while suggested optimal dietary levels for VK in juveniles are 1.5–20 mg VK3 kg^−1^ (reviewed in [11]). In fact, lower requirements might probably be due to the VK recycling system in hepatic and extra-hepatic tissues, through the conserved action of Vkorc1 and Vkorc1l1 along evolution [26].

Although no severe deformities (scoliosis, lordosis and/or kyphosis) were reported in Senegalese sole larvae when VK content in the commercial emulsion used to enrich Artemia was increased (up to 250 mg kg^−1^), a lower incidence of skeletal deformities per specimen, particularly at the caudal fin region, was observed [15]. Although those deformities of the caudal fin were small, they are largely accepted to be the source of the severe deformities encountered in the caudal fin (e.g., vertebral compression and/or fusion) in Senegalese sole at later stages, thus suggesting the former authors a slightly better osteological development when increased dietary VK content was offered to the larvae. In the same study, VK1 dietary supplementation for 40 days was associated with a lower expression of *ssvkorc1*. Here, expression of *ssvkorc1* increased when sole juveniles normally fed a diet containing 125 mg VK1 kg^−1^ were given a diet containing 10 times more VK1 for two days, but returned to basal (Control) values when the feeding schedule with supplemented VK1 diet was extended to five days. This result supports the hypothesis that VK recycling through ssVkorc1 and VK metabolism is regulated, and progressively adapts to altered VK1 dietary intake. In a short-term exposure (two days feeding), higher dietary VK content might lead to higher availability of VK, more γ-carboxylation reactions of VKDPs, and thus more VK epoxide. Similar to warfarin exposure, higher VK epoxide accumulation might require more VK recycling in order to avoid alterations of the redox status, resulting in an induction of higher expression of *vkorc1*. In a longer-term situation, different regulatory steps might be activated (e.g., blocking/reducing dietary VK assimilation at intestine through NPC1L1, or increased hydroxylation of VK1 by CYP4F2; reviewed in [10]) reducing the VK availability, and a lower expression of *vkorc1* might be restored. This was previously observed in Senegalese sole larvae fed increasing levels of VK for more than 30 days [15], and here after 5 days continuous feeding a diet containing 1250 mg kg^−1^. In contrast, expression of *ssvkorc1l1* remained unchanged in both conditions regardless of VK1 dietary intake, suggesting that a controlled metabolism and transport of K vitamers (VK1, VK2 metabolites, including menaquinone-4 and -7 (MK-4 and MK-7, respectively), and VK3) to extra-hepatic tissues might occur. Unfortunately, our understanding of how VK1 is absorbed at the intestinal lumen, transported or partially metabolized to VK3, and progressively prenylated to MK-4 by UbiA prenyltransferase domain-containing protein 1 (Ubiad1), is still limited (reviewed in [13]). Nevertheless, while VK1 is known to be preferentially retained in the liver to assist γ-carboxylation of clotting factors, VK2 has to be redistributed through circulation in order to be available for extra-hepatic tissues [84]. Moreover, while the low absorption efficiency of K vitamers from the diet (between 5% and 20% for VK1 and 55% for VK2 [85,86]) suggests a very high efficiency of hepatic VK recycling during the synthesis of blood clotting factors, it is still questioned whether similar efficiency is obtained in other tissues (e.g., bone and vessel wall), where the vkorc1l1 may recycle VK epoxides resulting from the extra-hepatic γ-carboxylation of VKDPs. Previous studies indicated that chemically induced VK deficiency (through warfarin exposure) leads to arterial calcification that could be rescued through VK2, but not VK1, dietary supplementation [87]. These observations reinforce the notion that fulfilling VK dietary requirements for normal development and physiological status may require both VK1 and VK2 sources. In any case, present and previous results [15,19], on how the two main VK pathways respond to increased/decreased dietary VK levels in the short/long term, suggest that evaluating gene expression of *ssvkorc1* or *sspxr*, but not *ssvkorc1l1*, might be suitable and reliable biomarkers of nutritional VK1 intake.

For animal health and development, fasting is one of the most drastic conditions determining their survival and growth potential. In this regard, although a one day fasting increases weaning success in Senegalese sole larvae, it also increases the incidence of skeletal deformities up to 88%, particularly at the neural arch, pleural and caudal vertebrae [88]. Here, a short fasting period of two days did not alter the expression levels of *ssvkorc1*, but it increased the expression of *ssvkorc1l1*, suggesting that extra-hepatic (but not the hepatic) γ-carboxylation of VKDPs, related to arterial uncalcification (Mgp; [89]) and bone mineralization (Bgp; [90]), may be compromised. Consequently, increased expression of both *sskvors* after three days re-feeding with a VK1 (1250VK) rich diet might be in line with: (i) increased dietary VK1 supplementation in the case of *ssvkorc1*, and a still unrecovered situation on extra-hepatic VK status regarding *ssvkorc1l1* gene expression, even when fed a VK1 rich diet; or (ii) the reported period of metabolic adjustments directed toward the physiological condition restoration after a fasting period [91]. Furthermore, similar transcriptional regulation of *ssvkorc1l1* and *sspxr* genes under fasting and re-feeding conditions (present study and [19]) evidenced a higher impact of fasting on specific roles of VK, particularly the transcriptional activation of Pxr signaling pathways and the extra-hepatic γ-carboxylation of VKDPs.

Warfarin is an anticoagulant drug commonly used at low doses for prevention and treatment of arterial and venous thromboembolic disorders in humans [92], but at high doses as a rodenticide, causing lethal hemorrhages in rats and mice, as well as other organisms including fish and birds [93,94]. Thus, it is considered an emerging contaminant negatively impacting the natural environment [95]. A recent review highlighted that the aquatic environment experiences a greater risk of anticoagulant rodenticide exposure than previously thought [96]. We and others have characterized and quantified the effects of warfarin exposure during fish embryogenesis, larval development and at adult stages [16,17,94], as well as described the particular molecular pathways altered in a global context [18]. We previously demonstrated that *sspxr* expression was altered upon warfarin exposure, but returned to normal values upon dietary VK1 supplementation [19]. A similar effect was observed for *ssvkorc1* and *ssvkorc1l1* under the same conditions, although *ssvkorc1l1* showed a higher up-regulation. Two different studies using in vitro cell culture models indicated that, in mammals, Vkorc1l1 is less sensitive to warfarin than Vkorc1 [24,27,28]. Although this might be in contradiction with our expression data, it is in agreement with the higher conservation of the warfarin binding (TYA) motif in ssVkorc1l1 than in ssVkorc1. Altered expression of *sspxr*, *ssvkorc1* and *ssvkorc1l1* upon warfarin exposure suggests that the known functions of VK—i.e., an activator of gene transcription and co-factor of hepatic and extra-hepatic Ggcx enzyme—may be affected upon anticoagulant exposure. Our data not only evidence the action of anticoagulants on VK-related gene expression; they highlight the risks that their presence in aquatic environments (in the water, sediment and/or organisms [97]) poses to aquatic animals, in particular to fish.

In conclusion, based on present and previous results, a combined analysis of *pxr*, *vkorc1* and *vkorc1l1* expression may be the best approach to assess the overall VK physiological status in Senegalese sole, while analysis of *pxr* and *vkorc1* expression should be sufficient to evaluate VK1 intake. Moreover, based on *vkors* gene expression profiles during larval development and at adult tissues, Senegalese sole might have higher dietary VK requirements during embryogenesis, pre- and pro-metamorphosis, and for proper gametogenesis than during juvenile stages. Therefore, future research efforts should be placed on determining VK content in embryos, live preys and inert diets (for weaning and breeding), and correlate them with important production traits such as survival, growth, quality and welfare, as well as with molecular data on VK pathways. Only with this approach we will be able to fine-tune the VK content in diets to promote Senegalese sole aquaculture.

## 4. Materials and Methods 

### 4.1. Ethics Statement

Fish facilities and persons in charge of animal experimentation were all accredited by the Portuguese National Authority for Animal Health (DGAV; permit 0421/000/000, 19 April 2016), and all experimental procedures involving animals followed the Animal Research: Reporting In Vivo Experiments (ARRIVE) guidelines, the European Directive 2010/63/EU, the related guidelines (European Commission, 2014) and the Portuguese legislation (Decreto-Lei 113/2013) for animal experimentation and welfare.

### 4.2. Rearing and Sampling Senegalese Sole Larvae and Juveniles

Senegalese sole eggs were obtained from natural spawning of fish broodstock at the Aquaculture Research Station of Pilot Fishing Station (EPPO)/ Instituto Português do Mar e da Atmosfera (IPMA) (Olhão, Portugal) and transferred to the fish facilities at the Centre of Marine Sciences (CCMAR), University of Algarve (Faro, Portugal). After thermal, light and chemical acclimatization, eggs were incubated in a semi-closed water recirculating system equipped with mechanical and biological filters, protein skimmer and a ultra violet (UV) sterilizer. After hatching, larvae were randomly distributed into four 100 L cylindro-conical tanks at a density of 95 larvae L^−1^. After settlement (21 days post-fertilization; dpf), post-larvae were transferred to 3-L flat bottom plastic trays (120 larvae per tray). Environmental parameters were: water temperature at 19.9 ± 1.2°C, water salinity at 36.9 ± 1.2 g L^−1^, dissolved oxygen saturation at 97.6% ± 4.9 %, a 12 h light/12 h dark photoperiod and 900 lux light intensity at water surface.

Larvae were progressively fed three times a day with live preys: rotifers (*Brachionus rotundiformis*) from 4 to 12 dpf, *Artemia* nauplii (AF strain; INVE, Salt Lake, Utah, USA) from 8 to 12 dpf and *Artemia* metanauplii (EG strain; INVE, Salt Lake, Utah, USA) enriched with Red Pepper^TM^ (Bernaqua, Olen, Belgium), supplemented with 250 mg VK1 kg^−1^ (as described in [15]) from 11 to 19 dpf. The same *Artemia* metanauplii, but immediately frozen after enrichment, were provided to the larvae from 20 dpf until the end of the experiment (32 dpf).

Fish previously euthanized with an overdose of tricaine methanesulfonate (MS-222, Sigma-Aldrich, Madrid, Spain) were sampled and washed with sterile distilled water. Embryonic samples were collected at 13.5, 18.5, 24, 36 (hatching) and 48 hours post-fertilization (hpf), while larval and juvenile samples were collected at 4, 7, 9, 11, 13, 15, 18, 22, 26 and 32 dpf. A total of 60 eggs, up to 20 larvae and 5 juveniles, depending on their size, were collected per replicate, kept in TRI-Reagent (Ambion, Alcobendas, Spain) and stored at −80 °C until processed for RNA extraction. 

### 4.3. Maintenance of Adult Senegalese Sole and Tissues Sampling

Adult fish from Aquaculture Research Station of EPPO/IPMA were transferred to the fish facilities of the CCMAR. After thermal, light and chemical acclimatization, and 15 days of maintenance in a semi-closed water recirculating system, fish were sacrificed and spleen, eye, brain, testis, ovaries, muscle, bone, skin, liver, heart, kidney, gills and intestine were sampled. Pools of tissues from 3 adults were placed in 10 volumes of TRI-Reagent and stored at −80 °C until processed.

### 4.4. Exposure of Senegalese Sole Juveniles to Different Physiological Conditions

RNA samples of Senegalese sole juveniles were obtained from an experiment conducted for a previous publication [19]. In brief, specimens aged 57 dpf (with 80 ± 7 mg wet weight (WW) and 14.91 ± 1.79 mm of standard length) grown under standard rearing procedures were distributed into 4 experimental flat bottom plastic trays (40 specimens per tray), and further cultured under the environmental conditions previously described. Juveniles were initially fed with 125 mg kg^−1^ DW VK1 inert diet (125VK1 diet) at 3% WW during 5 days, then: (i) fed with 125VK1 diet for 5 days (Control group); (ii) fasting during 2 days (Unfed group) and re-fed for 3 days with VK1 supplemented diet (1250 mg kg^−1^ DW, 1250VK1 diet; Re-fed group); (iii) fed with 125VK1 diet and exposed to 25 mg L^−1^ of warfarin (3-(α-acetonylbenzyl)-4-hydroxycoumarin sodium salt; Sigma-Aldrich, Madrid, Spain) during 2 days (Warfarin group) and re-fed for 3 days with 1250VK1 diet while still exposed to warfarin (Warfarin rescue group); or (iv) fed with 1250VK1 diet for 5 days (VK1 suppl group). Individual fish were sampled in triplicate from each experimental group at 2 and 5 days after each treatment initiated, euthanized with an overdose of MS-222, washed with sterile distilled water and stored in TRI-Reagent at −80 °C until RNA extraction.

### 4.5. RNA Isolation and Construction of Senegalese sole cDNA Library

RNA from larval and juvenile stages, and adult fish, tissues was prepared as described below. Aliquots of each RNA were pooled (to a total amount of 2 μg) and used to construct a cDNA library using the Marathon cDNA amplification kit (Clontech, Mountain View, CA, USA). Total RNA was converted into cDNA with oligo-(dT) and a special adaptor containing the two primers, AP1 and AP2, was attached to both ends of the cDNAs according to the manufacturer instructions. These marathon cDNA libraries were used as a template for the PCR reaction. 

### 4.6. cDNA Partial and Full-Length Amplification through 3′- and 5′-RACE

Degenerated primers used to amplify internal cDNA fragments of Senegalese sole *ssvkorc1* and *ssvkorc1l1* were designed in regions conserved among fish *vkor* sequences retrieved from GenBank and SoleaDB databases using on-site Basic Local Alignment Search Tool (BLAST) facilities. cDNA fragments of *ssvkorc1* and *ssvkorc1l1* were used to design gene-specific primers to amplify cDNA ends by RACE-PCR following the manufacturer instructions, and later the full-length cDNAs. PCR fragments were cloned in pCR2.1-TOPO TA cloning vector (Invitrogen, Alcobendas, Spain) and sequenced using CCMAR sequencing facilities. Gene-specific primers for the quantification of *ssvkorc1* and *ssvkorc1l1* relative gene expression by qPCR were designed. The sequences of the primers used in this work are presented in Table 1.

### 4.7. Sequences Collection and Phylogenetic Reconstruction

Vitamin K epoxide reductase (Vkors) protein sequences (54 in total; Table S1) were retrieved from GenBank database using on-site BLASTP tool with position-specific iterated BLAST (PSI-BLAST; [98]) algorithm. Sequences were aligned using MUSCLE software [99] and alignments were fed to MEGA version X to construct a molecular phylogeny [48]. Maximum Likelihood tree was created using a JTT model and a gamma distribution with site rate variation, and gaps were considered as partial deletion. For branching support, 500 bootstrap replications were analyzed. Vkors percentage identity matrixes were calculated using MUSCLE software. Sequence of the sea squirt (*Ciona intestinalis*) Vkor (NP_001073142) was used as outgroup.

### 4.8. Multiple sequence Alignment and Construction of Sequence Logos

Fish and tetrapod sequences for Vkorc1 and Vkorc1l1 were aligned using T-Coffee facilities [100]. Sequence alignments were improved by manual adjustments and fed to the Weblogo facilities [101] to construct taxon- and protein-specific sequence logos, where the height of each letter is directly proportional to its conservation, i.e., the more conserved residues are represented as larger characters.

### 4.9. RNA Extraction and qPCR Amplification

Total RNA was extracted from samples stored in TRI-Reagent following manufacturer instructions and purified using the High Pure RNA Isolation kit (Roche Applied Science, Alcobendas, Spain). RNA integrity was confirmed using Experion Automated Electrophoresis system (Bio-Rad, Alcobendas, Spain) and quantity was determined using NanoDrop spectrophotometer (Thermo Scientific, Alcobendas, Spain). Total RNA (1 µg) was reverse-transcribed for 1 h at 37 °C, using M-MLV reverse transcriptase (Invitrogen, Alcobendas, Spain), oligo-d(T) universal primer (5’-ACGCGTCGACCTCGAGATCGATG(T)_13_-3’) and RNase OUT (Invitrogen, Alcobendas, Spain). All quantitative real time PCR (qPCR) reactions were performed in triplicates using SsoFast EVAgreen Supermix (Bio-Rad), 0.25 μM of isoform-specific primers (Table 1) and 1:10 dilution of reverse transcribed RNA, in the StepOnePlus Real-Time PCR system (Applied Biosystems, Alcobendas, Spain). PCR amplification was as follows: an initial denaturation step of 1 min at 95 °C and 40 cycles of amplification (5 s at 95 °C and 10 s at 65 °C). A calibrator sample (cDNA pooled from all samples) was included in each qPCR plate [102]. Efficiency of amplification was between 96% and 104% for all primer sets. Levels of gene expression were calculated using the ΔΔCt comparative method and normalized using *ubiquitin* (*ubq*) RNA levels, a known reference gene for accurate normalization in qPCR studies with Senegalese sole [103]. Gene expression at 7 and 9 dpf for relative expression during development, while kidney and heart for relative expression in adult tissues were set to 1 and used as reference samples, for *ssvkorc1* and *ssvkorc1l1*, respectively.

### 4.10. Statistical Analysis

Results are given as mean and standard deviation. All data were checked for normality (Kolmogorov–Smirnov test) and homoscedasticity of variance (Bartlett’s test). Significant differences in gene expression were detected by one-way ANOVA or Student’s t-test. When significant differences were detected by one-way ANOVA, the Tukey multiple-comparison test was used to detect differences among experimental groups. Differences were considered to be significant when *P* < 0.05. Statistical analysis was done using Prims 5.0 (GraphPad Software, Inc.; San Diego, CA, USA).

## Figures and Tables

**Table 1:** Designed primers for initial amplification of Senegalese sole *vkors* cDNA sequences, full-length amplifications and relative gene expression.

Primer Name	Component	5’ to 3’ Nucleotide Sequences	Amplicon Size (bp)	Target Sequence
SsF1VKORC1L1-A_deg	Forward	agtgttcrgstcmaggtgggg	-	
SsR1VKORC1L1-A_deg	Reverse	aygtggtgaygcagatgaygc	-	
SsF2VKORC1L1-A_deg	Forward	ygtggagagggaamadgcbcgg	-	
SsR2VKORC1L1-A_deg	Reverse	ratvagrgcvgccatygcac	-	
SsF1VKORC1L1-B_deg	Forward	ccmgattacmgggcgmtgtgcg	-	
SsR1VKORC1L1-B_deg	Reverse	rcagaccatrcagaartchc	-	
SsF2VKORC1L1-B_deg	Forward	tggggacgwggwtttgghytgg	-	
SsR2VKORC1L1-B_deg	Reverse	gcytywgamacccaggaggm	-	
SsF2VKORmass-marathon	Forward	ccaggtgaaaccacagcgagcccc	-	
SsR2VKORmass-marathon	Reverse	tcccccaggtcacacatcgccc	-	
SsF1VKORc1l1-marathon	Forward	ccaacagtgtctatgggattgcttt	-	
SsR1VKORc1l1-marathon	Reverse	ctgaaaggcataaaaagcaatccca	-	
SsF2VKORc1l1-marathon	Forward	acatccatcttgtcggtggtgggt	-	
SsR2VKORc1l1-marathon	Reverse	agaggatgtagcccaggtagagtga	-	
SsVKORC1l1Fw3-Maraton	Forward	cggtggtgggttcactctacctgggc	-	
SsVKORC1l1Rev3-marathon	Reverse	gcccaagagaccaaaacctcgacccca	-	
SsVKORC1l1Fw4-marathon	Forward	actactgcgtcatctgcatcaccac	-	
SsVKORC1/massFw3-marathon	Forward	tgggggatttctgcgtggtctgcgt	-	
SsVKORC1/massRev3-marathon	Reverse	aggctgttgggctggttcagagggt	-	
SsVKORC1Fw2-qpcr	Forward	aaaccacagcgagcccctcc	245	KC108910
SsVKORC1Rev2-qpcr	Reverse	accgtttcattcatcaacaccacct		
SsVKORC1l1Fw2-qpcr	Forward	tggggttgtttcacgggcga	91	KC108911
SsVKORC1l1Rev2-qpcr	Reverse	ggacactctcaggacgggcg		

## Supplementary Materials

Supplementary materials can be found at https://www.mdpi.com/1422-0067/21/10/3489/s1.

## Abbreviations

ANOVA	Analysis of variance
BGP	Bone gla protein
BLAST	Basic Local Alignment Search Tool
bp	base pairs
cDNA	Complementary Deoxyribonucleic acid
DW	dry weight
GCCX	γ-glutamyl carboxylase (Ggcx)
hpf	hours post-fertilization
JTT	Jones-Taylor-Thornton
MEGA	Molecular Evolutionary Genetics Analysis
MGP	Matrix gla protein
MK-4	Menaquinone 4
MK-7	Menaquinone 7
M-MLV	Moloney Murine Leukemia Virus
M-MLV	Moloney Murine Leukemia Virus
MUSCLE	MUltiple Sequence Comparison by Log-Expectation
no	number
PCR	Polymerase Chain Reaction
PSI-BLAST	Position Specific Iterated Basic Local Alignment Search Tool
PXR	Pregnane X receptor
qPCR	Quantitative polymerase chain reaction
RACE	Rapid Amplification of cDNA Ends
RNA	Ribonucleic acid
SNPs	Single nucleotide polymorphisms
UBQ	Ubiquitin
UBUAD1	UbiA prenyltransferase domain-containing protein 1
VK1	Vitamin K1 - phylloquinone
VK2	Vitamin K2 - menaquinone
VK3	Vitamin K3 - menadione
VK	Vitamin K
VKDPs	Vitamin K dependent proteins
VKOR	Vitamin K epoxide reductase
VKORC1	Vitamin K epoxide reductase complex subunit 1
VKORC1L1	Vitamin K epoxide reductase complex subunit 1 like protein 1
VKORS	Vitamin K epoxide reductases
