# Supplementary material for: New Insights on Vitamin K Metabolism in Senegalese sole (Solea senegalensis) Based on Ontogenetic and Tissue-Specific Vitamin K Epoxide Reductase Molecular Data
Source: Int J Mol Sci. 2020 May 15;21(10):3489. doi: 10.3390/ijms21103489 (PMC7278968; doi:10.3390/ijms21103489)
Supplement: Supplementary file 1 [file ijms-21-03489-s001.zip › ijms-781028/Supplementary figure 1. VKORs-Solea senegalensis.docx]

**Supplementary figure 1.** Multiple-sequence alignment of Vkor proteins from sea squirt (*Ciona intestinalis*), Senegalese sole (*Solea senegalensis*), mouse (*Mus musculus*) and human (*Homo sapiens*).


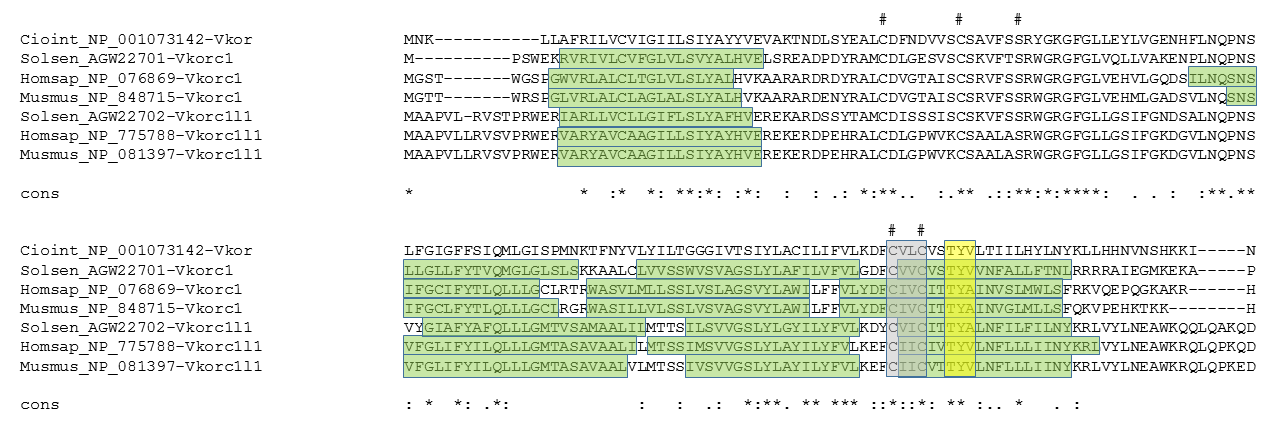


*Hash* at the top of the alignment indicates Vkor active sites (the four cysteines and the serine); the symbols *dot*, *colon* and *asterisk* at the bottom of the alignment reflect increasing conservation per residue in the aligned sequences; *yellow box*, warfarin binding motif (TYX); *gray box*, the known hydrophobic environment of the thiol redox site of the enzyme; *green boxes*, predicted transmembrane domains (TMhelix). Cioint, C. intestinalis; Solsen, S. senegalensis; Homsap, H. sapiens; Musmus, M. musculus. The multiple-sequence alignment was performed using T-Coffee facilities at tcoffee.org (Di Tommaso et al. 2011), and transmembrane domains were identified with TMhelix, Interproscan at EMBL-EBI (https://www.ebi.ac.uk/interpro/result/InterProScan/)
